# Galactocerebroside biosynthesis pathways of *Mycoplasma* species: an antigen triggering Guillain–Barré–Stohl syndrome

**DOI:** 10.1111/1751-7915.13794

**Published:** 2021-03-27

**Authors:** Erika Gaspari, Jasper J. Koehorst, Joachim Frey, Vitor A.P. Martins dos Santos, Maria Suarez‐Diez

**Affiliations:** ^1^ Laboratory of Systems and Synthetic Biology Wageningen University & Research Wageningen the Netherlands; ^2^ Vetsuisse University of Bern Bern Switzerland; ^3^ LifeGlimmer GmbH Berlin Germany

## Abstract

Infection by *Mycoplasma pneumoniae* has been identified as a preceding factor of Guillain–Barré–Stohl syndrome. The Guillain–Barré–Stohl syndrome is triggered by an immune reaction against the major glycolipids and it has been postulated that *M. pneumoniae* infection triggers this syndrome due to bacterial production of galactocerebroside. Here, we present an extensive comparison of 224 genome sequences from 104 *Mycoplasma* species to characterize the genetic determinants of galactocerebroside biosynthesis. Hidden Markov models were used to analyse glycosil transferases, leading to identification of a functional protein domain, termed M2000535 that appears in about a third of the studied genomes. This domain appears to be associated with a potential UDP‐glucose epimerase, which converts UDP‐glucose into UDP‐galactose, a main substrate for the biosynthesis of galactocerebroside. These findings clarify the pathogenic mechanisms underlining the triggering of Guillain–Barré–Stohl syndrome by *M*. *pneumoniae* infections.

## Graphical abstract

*Mycoplasma pneumoniae* is a preceding factor of Guillain‐Barré‐Stohl syndrome, triggered by an autoimmune reaction against a glycolipid named galactocerebroside, produced by the bacterium. In this work, we reconstruct the pathway and identify the functional protein domain responsible for the synthesis of galactocerebroside in Mycoplasmas.

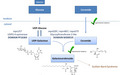

## Introduction

*Mycoplasma* species are bacteria representing the smallest free‐living organisms on earth. They include several pathogens infecting human, animals and plants. *Mycoplasma* is characterized by the lack of a cell wall, reason for which glycolipids of the membrane are exposed to the host’s immune system upon an infection. *Mycoplasma pneumoniae* is a human respiratory pathogen causing atypical (or walking) pneumonia, accounting for approximately 20–30% of all types of pneumonia worldwide (Liu *et al*., 2009; Varma‐Basil *et al*., 2009; Waites & Talkington, 2004; Zhang *et al*., 2016; Waites et al., 2017). Diagnosis of *M. pneumoniae* infections is currently performed mostly by PCR tests but remains complicated at an early stage of infection (Miyachi *et al*., 2009). Since about a decade, research to improve early diagnosis has focused on glycolipid antigens present on the membrane of *M. pneumoniae* (Matsuda, 2015). The percentage of glycolipids in *M. pneumoniae*’s membrane varies between 6% and 10% of total lipids (Gaspari et al., 2019).

For long, *M. pneumoniae* has been suspected as a potential preceding factor of Guillain–Barré–Stohl syndrome (GBS) (Ang *et al*., 2002; Yuki, 2007), which occurs at a frequency of about 5% of the total cases of past *M. pneumoniae* infections (van den Berg et al., 2014; Meyer Sauteur *et al*., 2016). The GBS is an autoimmune neurological disorder that is potentially life threatening. *Campylobacter jejuni* is the first microorganism that was associated with post‐infectious outbreak of GBS (Rees et al., 1995) and has been found to perform galactocerebroside biosynthesis (Hao *et al*., 1998). Galactocerebroside has been shown to be immunogenic to a low degree in *M. pneumoniae* infections (Kusunoki *et al*., 2001; Susuki *et al*., 2004), and it is postulated that *M. pneumoniae* triggers GBS by inducing anti‐galactocerebroside IgG (Meyer Sauteur et al., 2018; Smolders *et al*., 2019). In a clinical study about, a third of patients with central nervous system infections by *M. pneumoniae* revealed anti‐GalC antibodies indicating that *Mycoplasma pneumoniae* also might induce other CNS symptoms by other mechanisms (Christie et al., 2007).

Galactocerebroside, also called galactosylceramide, is a sphingolipid, more specifically a cerebroside, characterized by a galactosyl head group. A similar compound is glucocerebroside, alias glucosylceramide, which instead consists in a cerebroside where the monosaccharide head group is glucose. Glycosphingolipids such as galactocerebroside and glucocerebroside are typically synthetized in bacteria by the enzymes, ceramide galactosyltransferase (or galactosylceramide synthase – reaction EC 2.4.1.47) and ceramide glucosyltransferase (or glucosylceramide synthase – reaction EC 2.4.1.80) of the glycosyltransferase family. The enzyme ceramide galactosyltransferase appears in viruses and cellular organisms; it synthetizes the biosynthesis of galactocerebroside by binding UDP‐galactose to a ceramide molecule, releasing UDP.

The glycosyltransferase of *M. pneumoniae,* encoded by gene *mpn483,* has been shown to synthesize galactosylceramide (most likely the beta‐variant) using ceramide and UDP‐glucose as substrates, both with phosphatidylglycerol or cardiolipin as activators. *M. pneumoniae* has access to ceramide and galactose. It imports ceramide both from *in vitro* growth in axenic medium and from the host *in vivo* during infection (Klement *et al*., 2007). In addition, it can use the fatty acid chains from incorporated ceramide in other lipids to build up ceramide‐based glycolipids. Moreover, *M. pneumoniae* favours the import of glucose *in vivo*, albeit not *in vitro,* in which galactose is preferred (Plackett *et al*., 1969). Finally, it is postulated but not confirmed that *M. pneumoniae* contains a potential epimerase converting UDP‐glucose into UDP‐galactose (Dandekar *et al*., 2000).

Characterization of the galactocerebroside biosynthesis pathway in *M. pneumoniae* will further clarify pathogenic mechanisms and can greatly impact the development of methods early detection and diagnosis of GBS. Moreover, these data are essential for the design of alternative metabolic pathways for *M. pneumoniae* avoiding galactocerebroside formation and to identify alternative *Mycoplasma* species devoid of galactocerebroside for biomedical applications.

We present here a comparative analysis investigation of 9 strains of *M. pneumoniae* and additional 103 currently genome‐sequenced *Mycoplasma* species. The goal is to identify the genetic determinants of galactocerebroside biosynthesis and to further characterize the proteins in this pathway.

## Experimental procedures

All available and complete *Mycoplasma* genome sequences were retrieved from the NCBI Genome repository. Overall, 224 genome sequences were obtained, belonging to 104 species that are listed in File S1. All genome sequences were re‐annotated using the SAPP pipeline (Koehorst *et al*., 2017). Gene prediction was performed using Prodigal 2.6.3 (Hyatt *et al*., 2010), and protein sequences were annotated using InterProScan 5.36.75.0 (Jones *et al*., 2014) to assign functional domains. Annotation data were stored in a triple‐store (GraphDB) (Güting, 1994) in a linked data format using the GBOL ontology as schema (van Dam *et al*., 2019) and systematically queried using SPARQL.

Hidden Markov Models (HMMs) were built and searched for with HMMER v3.3 (Eddy, 1998) – through commands *hmmbuild* and *hmmsearch* – on multiple sequence alignments (MSAs) performed with Clustal Omega 1.2.4 (Sievers *et al*., 2011; Madeira *et al*., 2019).

Sequence logos have been generated with WebLogo version 3 (Crooks et al., 2004).

## Results

We studied the functionalities associated with the *M*. *pneumoniae* genome, through an analysis of protein domains, to describe the biosynthesis pathway that leads to the formation of galactocerebroside and compare it to the pathways found in other mycoplasmas. Therefore, we analysed 224 genomes of 104 *Mycoplasma* species (given in File S1), comprising 213 strains. To ensure uniform annotation and a consistent comparison, all genomes were re‐annotated.

The synthesis of glycosphingolipid such as galactocerebroside, in bacteria, needs a glycosyltransferase enzyme, linking a sugar (galactose) to a phospholipid (ceramide) and building the glycosyl bond. Our functional analysis identified 4 genes containing a glycosyltransferase domain in *M. pneumoniae*. These have been found in the 9 analysed strains and correspond to locus tags *mpn028, mpn483, mpn075* and *mpn064* in *M. pneumoniae* M129. While *mpn064* (*deoA*) codes for a thymidine phosphorylase (EC 2.4.2.2) that contains a ‘glycosyl transferase family 3’ domain with InterPro identifier IPR000312, *mpn028, mpn483* and *mpn075* contain a ‘glycosyltransferase 2‐like’ domain, ( IPR001173), associated with proteins that have been linked to glycosphingolipids biosynthesis pathways and, specifically, to proteins that have been proven to own glycosyltransferase activity (Sobhanifar *et al*., 2016). Their lengths and E‐values of associated glycosyltransferase are shown in Table 1. The glycosyltransferase with more significant E‐value is MPN_028, while the one known to synthetize galactocerebroside is MPN_483 (Klement *et al*., 2007).

**Table 1:** The three enzymes MPN_028, MPN_075 and MPN_483 of *Mycoplasma pneumoniae* M129 matching the InterPro domain IPR001173 (“Glycosyltransferase 2‐like”), with E‐values and gene length.

*Mycoplasma pneumoniae* M129 Enzyme	E‐value: glycosyltransferase 2‐like domain	Gene length (bp)
MPN_028	2.7 × 10^‐26^	899
MPN_483	1.2 × 10^‐25^	1025
MPN_075	1.2 × 10^‐21^	899

**Fig. 1:**
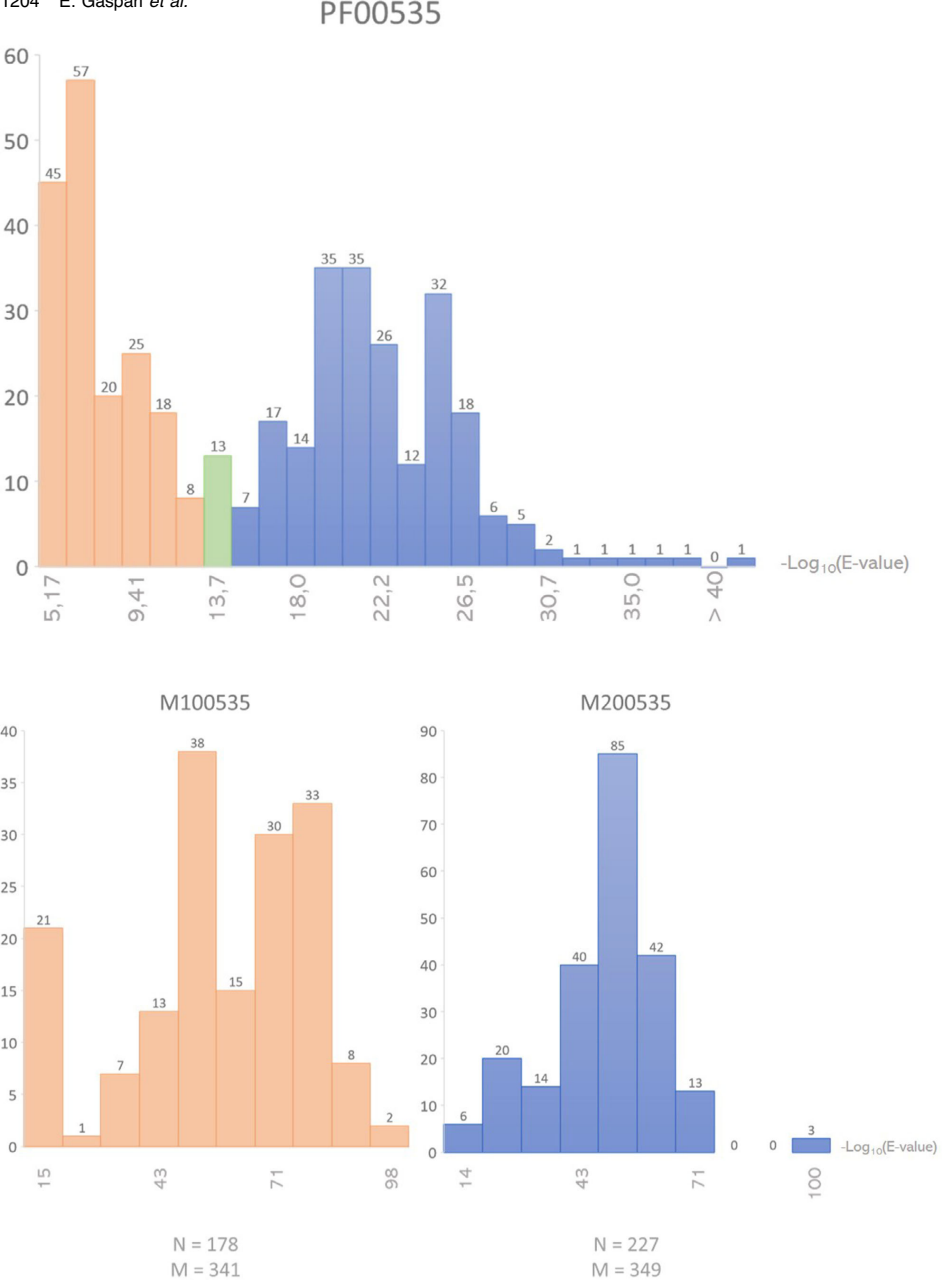
Top centre) Distribution of number of expected hits of all the *Mycoplasma* protein sequences to the Pfam domain PF00535 (‘Glyco_transf_2’). Expected hits are reported with correspondent ‐Log_10_(E‐value). The histogram shows two different peaks: new HMMs were built on the two groups of sequences. The first group, ‐Log_10_ (E‐value) lower or equal to 13.4, is marked in orange and contains 178 sequences and the second group, ‐Log_10_(E‐value) higher or equal to 13.6, is marked in blue and contains 227 sequences. The 4 sequences with ‐Log_10_(E‐value) equal to 13.5 (comprised in the green bar) are used in both groups. Lower) distribution of matches to the two new motifs. The two new motifs obtained are called M100535 (left) and M200535 (right) and have amino acids lengths M = 341 and M = 349 respectively.

The InterPro IPR001173 domain comprises two Pfam domains: ‘Glyco_trans_2_3’ (PF13632) and ‘Glyco_transf_2’ (PF00535); this last is found in two glycosyltransferases of *C. jejuni*, with E‐values of 10^‐29^ (Putative galactosyltransferase – UniProtKB Q8KWR2) and 10^‐27^ (Beta‐1,3‐galactosyltransferase coded by gene *cgtB* – UniProtKB Q5DT13). Focusing on the *Mycoplasma* species, we therefore assumed the protein sequences containing the Pfam domain PF00535 to be the ones associated with galactocerebroside synthesis. Thus, we continued our analysis focusing on PF00535 and discarded further analysis of PF13632. The distribution of the E‐values of this signature, presented in Fig. 1, shows a bimodal distribution. The presence of two peaks suggests two similar but distinct domains. Thus, we re‐built HMMs on the two separate groups obtaining two new domains, M100535 and M200535. Then, E‐values for these domains on the sequences were re‐calculated, as indicated in Fig. 1. This approach results in two groups of protein sequences each matching its corresponding motif with much higher significance, that is much lower E‐values. All the strains of *M. pneumoniae* contain only the second motif M200535. The HMM for M200535 motif is provided in File S2. The two glycosyltransferases of *C. jejuni* match M200535 with much higher significance than PF00535: the beta‐1,3‐galactosyltransferase with E‐value 10^‐43^ and putative galactosyltransferase with E‐value 10^‐42^. An additional glycosyltransferase family 2 protein matching M200535 with E‐value 10^‐42^ was identified in *C. jejuni,* and full results are given in File S3.

**Fig. 2:**
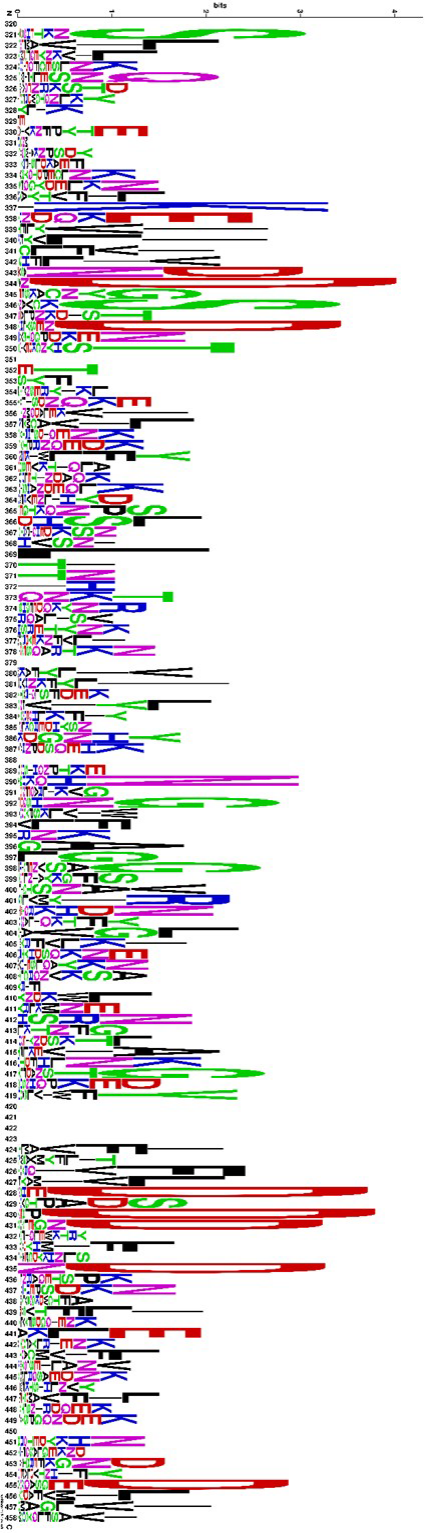
Sequence logo of protein domains M200535 from sequence position 320 to 458. M200535 is abundant in aspartic acid (D) in positions 344, 348, 428, 430, 436 and 455.

The two new M100535 and M200535 motifs show some substantial differences: the sequence alignments of the proteins carrying the domains reveal M200535 to be almost a twice as long as M100535. Most differences between the two motifs are present in the first part of the domain, where M200535‐containing sequences show a predominance of aspartic acid in positions 344, 348, 428, 430, 436 and 455 (Fig. 2). Consensus sequences for both M100535 and M200535 are provided in File S4.

Occurrences of the domains M100535, M200535, here defined, and of domains PF00534, PF13439 or PF13641 associated with alternative glycosyltransferases are summarized in Table 2. In total, 73 out of the 104 *Mycoplasma* species analysed match at least one glycosyltransferase domain. It should be noted that any genome containing either M100535 or M200535 also contains PF00535.

**Table 2:** Number of glycosyltransferases found in each *Mycoplasma* species analysed for domains M100535, M200535, PF00534, PF13439 and PF13641.

Species	N. glycosyltransferases found with domain				
	M100535	M200535	PF00534	PF13439	PF13641
*M. agalactie* ^a^	1	1/2	0	0	1
*M. agassizii*	1	0	1	0	0
*M. alligatoris*	1	2	0	0	1
*M. alvi*	5	3	5	0	2
*M. amphoriforme*	0	5	1	0	0
*M. anatis*	1	1	0	0	0
*M. anseris*	1	0	0	0	0
*M. arginini*	1	0	0	0	0
*M. arthritidis*	1	0	0	0	0
*M. bovigenitalium*	2	2	1	1	2
*M. bovirhinis*	1	0	0	0	0
*M. bovis*	1	1	0	0	1
*M. bovoculi*	0	2	0	0	0
*M. buteonis*	1	0	0	0	0
*M. californicum* ^a^	1/2	1	1	1	2
*M. canadense*	1	0	0	0	0
*M. canis*	1	0	0	0	0
*M. capricolum*	1	0	0	0	1
*M. cloacale*	1	0	0	0	0
*M. collis*	1	1	1	0	0
*M. colombinum*	1	0	0	0	0
*M. columborale*	1	0	0	0	0
*M. conjunctivae*	0	1	1	1	0
*M. cricetuli*	1	0	0	0	0
*M. crocodyli*	1	2	0	0	1
*M. cynos*	1	0	0	0	0
*M. elephantis* ^b^	2/3	2/3	0	0	0
*M. falifaucium*	1	0	1	1	0
*M. felis*	1	0	0	0	0
*M. fermentans*	1	1	1	1	1
*M. gallinarum*	1	1	0	0	1
*M. gallisepticum*	1	1	0	0	0
*M. gallopavonis*	1	0	0	0	0
*M. genitalium* ^a^	1	2/3/4	0	0	0
*M. girerdii*	2	0	0	0	0
*M. glycophylum*	1	0	0	0	0
*M. hyorhinis*	1	0	0	0	0
*M. imitans*	0	1	0	0	0
*M. iners*	1	0	0	0	0
*M. iowae*	2	0	0	0	1
*M. leachii*	1	0	0	0	0
*M. leonicaptivi*	1	0	0	0	0
*M. lipofaciens*	1	1	0	0	0
*M. melagridis*	1	0	0	0	0
*M. moatsii*	2	6	2	0	1
*M. mobile*	1	2	1	0	0
*M. molare*	1	0	0	0	0
*M. mustelae*	1	0	0	0	0
*M. mycoides*	1	2	0	0	3
*M. penetrans*	0	3	0	0	1
*M. phocidae*	1	0	0	0	0
*M. phocirhinis*	1	0	1	1	0
*M. pirum*	2	9	4	1	1
*M. pneumoniae*	0	3	0	0	0
*M. primatum*	0	3	0	0	0
*M. pullorum*	1	0	0	0	0
*M. pulmonis*	1	0	0	0	0
*M. putrefaciens*	1	0	0	0	0
*M. simbae*	1	0	1	1	0
*M. sp. 5H*	0	3	1	0	0
*M. sp. Bg1*	0	0	0	0	0
*M. sp. CAG:472*	1	1	2	3	0
*M. sp. CAG:611* ^a^	0	2/3	3	2	1
*M. sp. CAG:776*	1	9	3	2	2
*M. sp. CAG:877*	1	6	5	4	0
*M. sp. CAG:956*	3	7	2	1	1
*M. sp. G5847*	3	1	0	0	1
*M. sp. PE*	1	2	1	0	0
*M. sp. UBA710*	2	4	0	0	0
*M. sturni*	1	0	0	0	0
*M. subdolum*	1	0	0	0	0
*M. synoviae*	1	0	0	0	0
*M. testudinis*	2	7	4	0	0
*M. yeatsii*	1	0	0	0	0

^a^ The number of glycosyltransferases among the different strains of the same species, else the same number must be considered for all the strains of the indicated species.

^b^ One of the glycosyltransferase domains matches both M100535 and M200535.

*In silico* functional analysis on *M. pneumoniae* reveals that *mpn257* contains Pfam domain ‘GDP‐mannose 4,6 dehydratase’ (PF16363) comprised in the InterPro domain IPR016040 (E‐value: 2.4 10^‐46^), found in sequences annotated as UDP‐6‐glucose 4‐epimerases that interconvert UDP‐glucose into UDP‐galactose. Therefore, we assume *M. pneumoniae* has the ability of converting UDP‐glucose into UDP‐galactose.

**Fig. 3:**
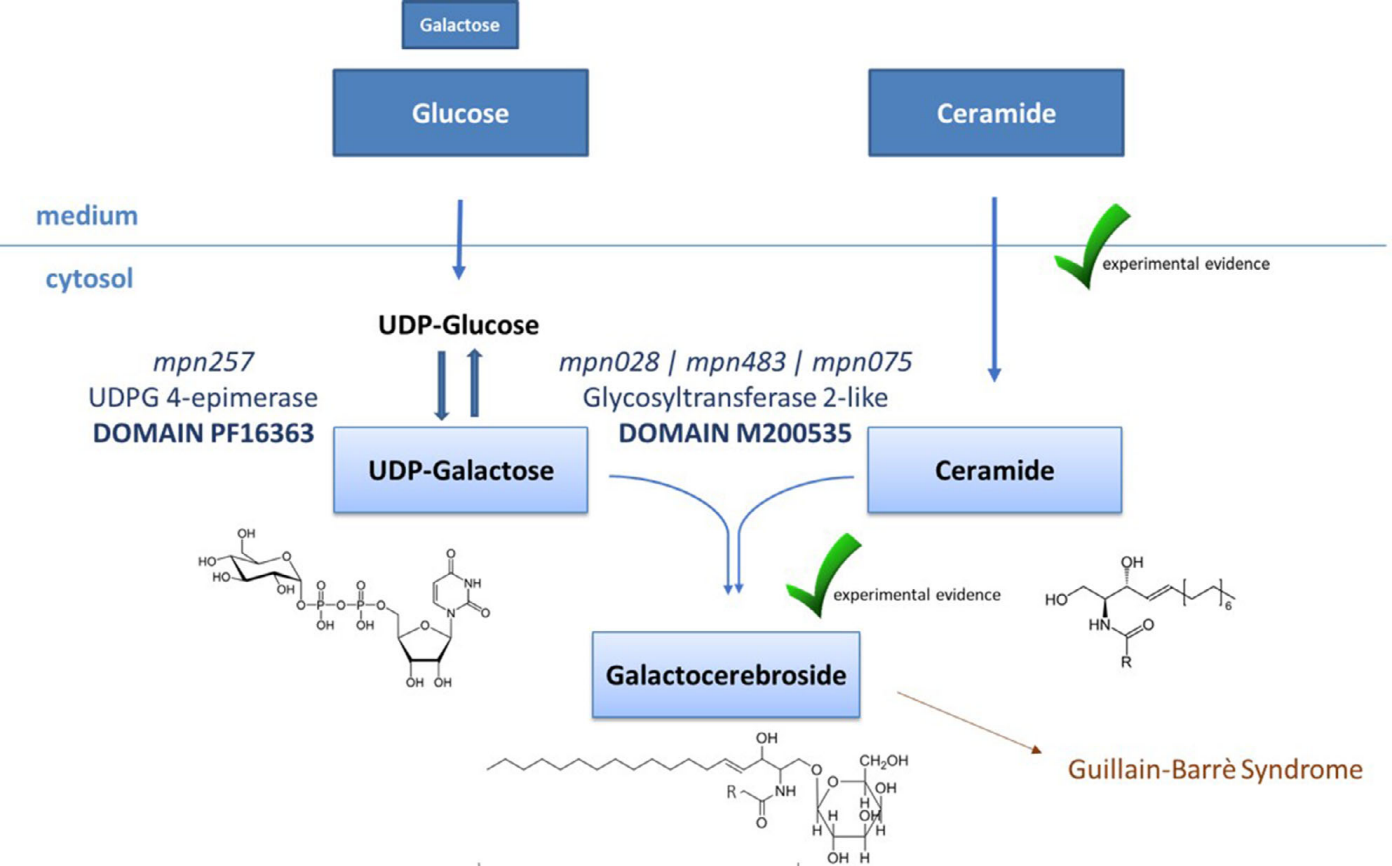
In vivo galactocerebroside biosynthesis pathway in *Mycoplasma pneumoniae* according to functional analysis. *M. pneumoniae* favours the import on glucose over galactose in vivo. Once in the cytosol, UDP‐glucose in converted into UDP‐galactose by the epimerase containing the functional domain PF16363, encoded by gene *mpn257*. UDP‐galactose is then used by one of the glycosyltransferases encoded by *mpn028*, *mpn483* or *mpn075*, all containing the functional domain M200535. The import of ceramide and the synthesis of galactocerebroside are supported by experimental evidences.

We can conclude the pathway for galactocerebroside synthesis in *Mycoplasma pneumoniae in vivo* is most likely as represented in Fig. 3.

**Fig. 4:**
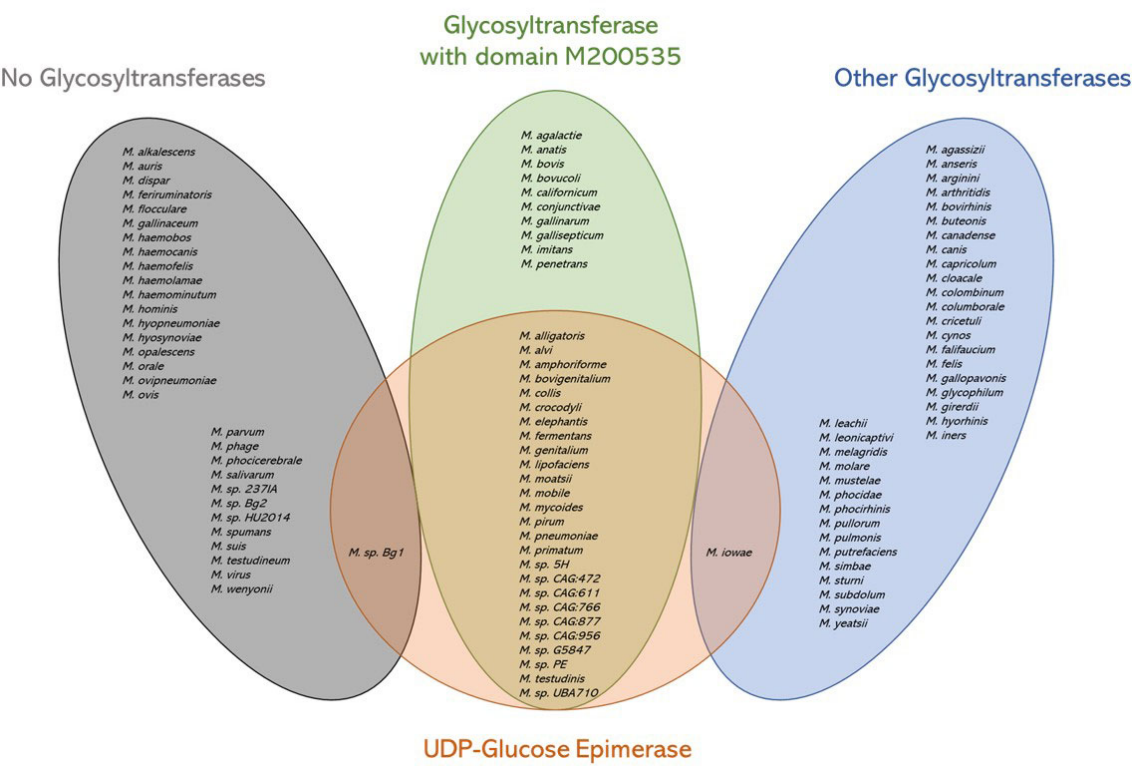
Venn diagram of the three glycosyltransferase‐representing groups of *mycoplasmas* (no glycosyltransferase – 31 species – in grey, at least one glycosyltransferase with domain M200535 – 36 species – in green, other glycosyltransferases with domains different from M200535 – 37 species – in blue) intersecting the group of Mycoplasmas containing at least one UDP‐glucose epimerase (28 species – in red). Except for *M. sp. Bg1* and *M. iowae*, only species with M200535 show presence of UDP‐glucose epimerase (26 species).

Domain Pfam PF01370 is associated with a functionally equivalent epimerase. Interestingly, analysis of all *Mycoplasma* species shows that almost only the species containing at least one domain, M200535 matched the UDP‐glucose‐epimerase domains Pfam PF16363 and/or PF01370 (Fig. 4). Exceptions are *M*. sp. *Bg1* (not containing any glycosyltransferase domain but matching an epimerase domain) and *Mycoplasma iowae* (containing a glycosyltransferase domain different from M200535).

## Discussion

Our results show that all strains of *M. pneumoniae* analysed have at least three glycosyltransferases encoded in the genome (in *M. pneumoniae* strain M129 these are MPN_075, MPN_028 and MPN_483) that can potentially perform the synthesis of galactocerebroside. The highly significant match of M200535 with *C. jejuni* galactosyltransferases indicates that the sequences responsible of galactocerebroside synthesis contain the functional domain M200535, which, in this study, was found to be a distinctive motif with specific feature, while previously included in the Pfam domain PF00535. The *Mycoplasma* species and strains containing at least a protein with the functional domain M200535 are the only *Mycoplasma* (with the two exceptions *M. iowae* and the taxonomically not yet defined *M*. sp. Bg1) showing concomitant presence of a UDP‐glucose epimerase domain, which converts UDP‐glucose into UDP‐galactose. UDP‐galactose is used in the galactocerebroside synthesis and favoured, over UDP‐glucose, as a substrate by the glycosyltransferase operating the linkage. In fact, M200535 contains conserved aspartic acid residues, which in other microorganisms such as *E. coli* have been found to be catalytically essential for glycosyltransferase to exploit their β‐transferase activity on UDP‐sugars (Griffiths et al., 1998).

Our analysis pinpoints that almost all the *Mycoplasma* species infecting humans contain the domain M200535 (Fig. 4). Other *Mycoplasma* species without the functional domain M200535 show an absence of UDP‐glucose epimerase. About half of them lack any domain associated with glycosyltransferase function; therefore, this set of species would be most likely unable to synthesize galactocerebroside that triggers GBS. Among the human‐related *Mycoplasma* species, those with no glycosyltransferase domain are *M. hominis* and *M. orale*, while among respiratory tract‐related species, present in organisms other than humans, are the calves’ *M. dispar*, the swine’s *M. flocculare* and *M. hyopneumoniae*, the caprine´s *M. ovipneumoniae*, dog´s *M. spumans* and turtle’s *M. testudineum*. The rest of the species without functional domain M200535, and therefore lacking UDP‐glucose epimerase, comprise *Mycoplasma* species with one or more glycosyltransferase enzymes containing domains. Glycosyltransferases with domains such as M100535 do not show conserved presence of aspartic acid residues, indicating they might be unable to use UDP‐sugars as substrate and, in consequence, to perform the synthesis of galactocerebroside. Human *Mycoplasma* species with glycosyltransferases lacking the domain M200535 have not been detected, while the following non‐human respiratory tract‐related species have been identified with glycosyltransferase and absence of M200535: tortoise´s *M. agassizii*, domestic animal´s *M. arginine*, bovine *M. bovirhinis* and *M. canadense*, wild bird´s *M. buteonis*, goat´s *M. capricolum*, pigeon´s *M. columborale*, canine *M. cynos*, pig´s *M. hyorhinis*, chicken´s *M. iners* and *M. synoviae*, turkey´s *M. melagridis*, dog´s *M. molare*, mink´s M. mustelae, seal´s *M. phocidae* and *M. phocirhinis*, avian *M. pullorum* and lion´s *M. simbae*.

Our work leads to the suggestion of genetic modifications that would validate the hypotheses formulated by computational analysis. The characterization of the pathway indeed suggests which genes should be a primary target for genetic modifications to avoid biosynthesis of galactocerebroside in *M. pneumoniae*. The most intuitive strategy would be the knock‐out of the genes encoding for the glycosyltransferases blocking the transfer of galactose to ceramide. However, this seems to be non‐trivial: in a global transposon mutagenesis inactivation experiment of *M*. *genitalium,* the gene encoding for the glycosyltransferase MG_517, homologous of MPN_483 in *M. pneumoniae* (Klement *et al*., 2007), remained untouched suggesting the gene encoding for this enzyme is essential (Glass *et al*., 2006). Although it is proven that *mg517* and *mpn483* share 77% of gene sequence similarity, the enzymatic activities slightly differ in terms of specificity (Andrés, 2011). The essentiality of *mpn483*, not excluded by gene transposon analysis (Lluch‐senar et al., 2015), is expected as *M. pneumoniae* uses this enzyme to perform synthesis of many other lipids in the membrane (Klement, 2007), which we know are crucial for its survival (Gaspari *et al*., 2020). In the same way, the gene transposon analysis conducted by Lluch‐Senar et al. suggests *mpn075* and *mpn257*, respectively, coding for the glycosyltransferase MPN_075 and the epimerase MPN_257, might be essential, at the contrary of *mpn028*, coding for the third glycosyltransferase, which is reported to be non‐essential. A validation of our computational analysis would consist in knocking out *mpn028* and replacing *mpn483* and *mpn075* with genes coding for glycosyltransferases that do not possess the motif M200535. To facilitate the genetic modification, genes coding for glycosyltransferases of other mycoplasmas should be chosen, among the ones not containing the motif M200535 (Fig. 4). However, the essentiality of genes *mpn075* and *mpn257* remains to be clarified, due to the uncertainty of the gene transposon analysis method in establishing gene essentiality. Moreover, the essentiality of the gene arises only after several strain passages (Lluch‐Senar *et al*., 2015). The knock‐out of *mpn257*, coding for the UDP‐glucose epimerase, would indeed constitute and additional layer of safety: the limited import of galactose *in vivo* is not a sufficient condition for assuming limiting quantities for biosynthesis of galactocerebroside, as the bacterium could take the needed amount of galactose from glucose conversion.

*M. pneumoniae* is the *Mycoplasma* species which metabolome, transcriptome and proteome are among the best‐studied, hence, giving access to profound basic research opportunities and development of novel approaches in biotechnology and biomedicine (Yus *et al*., 2009; Maier *et al*., 2013; Chen *et al*., 2016; Trussart *et al*., 2017; Yus et al., 2019), among which live attenuated vaccines (www.mycosynvac.org) and respiratory tract‐related live biotherapeutics (www.pulmobio.com). Therefore, an experimental application of our computational results would represent a suitable approach to render this bacterium safe in its numerous applications. In fact, other attempts to avoid biosynthesis of galactocerebroside seem to be not trivial: it would not be possible to knock‐out genes involved in sphingolipid transporter since this transporter would not only import ceramide but also very important lipids such as sphingomyelin that were shown to be essential to the survival of *M. pneumoniae* (Gaspari *et al*., 2020). Instead, the introduction of a ceramidase, disassembling ceramide into its sphingosine backbone and fatty acids chains, is another potential strategy to integrate. In bacteria, this enzyme has been found in *Pseudomonas aeruginosa* (Kita et al., 2000; Okino et al., 1998) and *Mycobacterium tuberculosis* (Okino et al., 2010) to be neutral (Tani et al., 2004) and reversible (Ito et al., 2014), so not always favouring degradation of ceramide but also its synthesis, according to the environmental and cytosolic conditions. However, *M. pneumoniae* affinity for sphingomyelin is unique, therefore, the degradation of ceramide into sphingosine and fatty acids could be of advantage for *M. pneumoniae* to build up sphingomyelin, which it typically imports unchanged from the medium (Worliczek *et al*., 2007). The *in silico* characterization of the protein domain that might be responsible of the galactocerebroside biosynthesis has potential impact as a target for drugs related to post‐infectious GBS: the elucidation of the pathway and its analysis on mycoplasmas could be used to assess the risk of post‐infectious GBS development, helping in the development of therapeutical strategies for early diagnose and/or control of GBS.

Moreover, in this manuscript we report a group of mycoplasmas that lacks both UDP‐glucose epimerase, converting UDP‐glucose into UDP‐galactose, and any glycosyltransferase domain, therefore unable to complex lipids with sugars. This group consists of *M. alkalescens, M. auris, M. dispar, M. feriruminatoris, M. flocculare, M. gallinaceum, M. haemobos, M. haemocanis, M. haemofelis, M. haemolamae, M. haemominutum, M. hominis, M. hyopneumoniae, M. hyosynoviae, M. opalescens, M. orale, M. ovipneumoniae, M. ovis, M. parvum, M. phage, M. phocicerebrale, M. salivarum, M. spumans, M. suis, M. testudineum, M. virus, M. wenyonii* and the uncharacterized Mycoplasma species named 237IA, Bg2 and HU2014. Lacking the whole machinery for galactocerebroside synthesis, this group of Mycoplasma species may be considered suitable for biomedical applications in terms of reducing the risk of post‐infectious GBS.

## Conclusion

All *Mycoplasma pneumoniae* strains have genes encoding for glycosyltransferases, of which at least two are essential and at least one has been proved to encode for an enzyme (MPN_483 in M129) that can synthetize glycosphingolipids such as galactocerebroside. Most likely, MPN_028 and MPN_075 perform the same synthesis, as they show high significant match for motif M200535. This motif was found as well in *C. jejuni,* the first microorganism linked to GBS through galactocerebroside biosynthesis. While the access of *M. pneumoniae* to galactose *in vivo*, when the import of glucose is at higher rate, remains unclear, the presence of a UDP‐glucose epimerase MPN_257, converting UDP‐glucose into UDP‐galactose, could make the synthesis of galactocerebroside possible. We can conclude that all wild‐type strains of *M*. *pneumoniae* are potentially capable of synthetizing galactocerebroside and will most likely be able to do so even with substitution or knock‐out of the galactosyltransferase MPN483 or the epimerase MPN_257, which will be problematic for certain medical applications, that is human vaccines and live biotherapeutics. Similarly, this will be the case for *Mycoplasma* species with the functional domain M200535, presenting also a UDP‐glucose epimerase domain. Our data show a set of *Mycoplasma* species that could serve as alternatives for such biomedical applications or that could provide pathway genes to modify *M. pneumoniae* accordingly.

## Conflict of interest

Patent application n. EP20174842.3 by the Wageningen University & Research. Author Vitor A.P. Martins dos Santos has interests in the company LifeGlimmer GmbH. All authors declare that the research was conducted in the absence of any commercial or financial relationships that could be construed as a potential conflict of interest.

## Author contributions

JF suggested the study. EG, JJK, JF, MS‐D and VAPMdS conceived the project. JJK performed the functional domain analysis. EG developed the motif model. EG wrote the manuscript with input of MS‐D, JF, VAPMdS and JJK. JF, MS‐D and VAPMdS supervised the project. All authors read and approved the manuscript.

## Supporting information


**File S1.** List of *Mycoplasma* genome sequences used in the study, obtained from NCBI repository.

**File S2.** Motif M200535, suggested to be responsible for galactocerebroside biosynthesis, as Hidden Markov Model.

**File S3.** Result of HMM search of motif M200535 in *Campilobacter jejuni*.

**File S4.** Consensus sequences for motifs M100535 and M200535.

## Data Availability

The authors declare that all the data supporting the modelling are available within the paper and its supplementary information.
