# Supplementary material for: Galactocerebroside biosynthesis pathways of Mycoplasma species: an antigen triggering Guillain–Barré–Stohl syndrome
Source: Microb Biotechnol. 2021 Mar 27;14(3):1201–11. doi: 10.1111/1751-7915.13794 (PMC8085918; doi:10.1111/1751-7915.13794)
Supplement: Supplementary file 5 [file MBT2-14-1201-s005.docx]

Supplementary Material

# Supplementary File 1

List of *Mycoplasma* genome sequences used in the study, obtained from NCBI repository. The 224 genome sequences belong to 104 *Mycoplasma* species and 213 *Mycoplasma* strains.

# Supplementary File 2

Motif M200535, suggested to be responsible for galactocerebroside biosynthesis, as Hidden Markov Model.

## Supplementary File 3

Result of HMM search of motif M200535 in *Campilobacter jejuni*.

## Supplementary File 4

Consensus sequences for motifs M100535 and M200535.
